# Impact of coronavirus disease 2019 on patients with primary adrenal insufficiency: a cross-sectional study

**DOI:** 10.1530/EC-23-0122

**Published:** 2023-07-12

**Authors:** Gregory Knowles, Emily Warmington, Lisa M Shepherd, Jonathan M Hazlehurst, Anne de Bray, Helena Gleeson, Wiebke Arlt, Alessandro Prete

**Affiliations:** 1 Walsall Manor Hospital, Walsall, UK; 2 College of Medical and Dental Sciences, University of Birmingham, Birmingham, UK; 3 Department of Endocrinology, University Hospitals Birmingham NHS Foundation Trust, Birmingham, UK; 4 Institute of Metabolism and Systems Research, University of Birmingham, Birmingham, UK; 5 Institute of Applied Health Research, University of Birmingham, Birmingham, UK; 6 Medical Research Council London Institute of Medical Sciences, London, UK; 7 NIHR Birmingham Biomedical Research Centre, University of Birmingham and University Hospitals Birmingham NHS Foundation Trust, Birmingham, UK

**Keywords:** COVID-19, SARS CoV 2, coronavirus, Addison’s disease, congenital adrenal hyperplasia, adrenal insufficiency, surveys and questionnaires

## Abstract

**Objective:**

Patients with primary adrenal insufficiency (PAI) are thought to be particularly vulnerable to coronavirus disease 2019 (COVID-19); however, little is known about its true impact on this group. We assessed morbidity and health promotion attitudes during the pandemic amongst a large cohort of patients with PAI.

**Design:**

Cross-sectional, single-centre study.

**Methods:**

In May 2020, COVID-19 advice on social distancing and sick-day rules was distributed to all patients with PAI registered with a large secondary/tertiary care centre. A semi-structured questionnaire was used to survey patients in early 2021.

**Results:**

Of 207 contacted patients, 162 responded (82/111 with Addison’s disease, AD; 80/96 with congenital adrenal hyperplasia, CAH). Patients with AD were older than those with CAH (median age 51 vs 39 years; *P* < 0.001) and had more comorbidities (Charlson comorbidity index ≥2 47.6% vs 10.0%; *P*< 0.001). By the time of the survey, 47 patients (29.0%) had been diagnosed with COVID-19, the second commonest cause of sick-day dosing during the study and the leading trigger of adrenal crises (4/18 cases). Patients with CAH had a higher risk of COVID-19 compared to AD (adjusted odds ratio 2.53 (95% CI 1.07–6.16), *P*= 0.036), were less inclined to have the COVID-19 vaccine (80.0% vs 96.3%; *P* = 0.001), and were less likely to have undergone hydrocortisone self-injection training (80.0% vs 91.5%; *P* = 0.044) or wear medical alert jewellery (36.3% vs 64.6%; *P* = 0.001).

**Conclusions:**

COVID-19 was a principal trigger for adrenal crises and sick-day dosing in patients with PAI. Despite a higher risk of COVID-19, patients with CAH showed less engagement with self-protective attitudes.

**Significance statement:**

We conducted a cross-sectional study on a large and well-characterised group of patients with PAI and demonstrated that COVID-19 was a leading cause of morbidity during the early phases of the pandemic. Patients with AD were older and had a greater burden of comorbidity than those with CAH, including non-adrenal autoimmune disorders. However, patients with CAH were more likely to develop COVID-19 and demonstrated reduced engagement with healthcare services and health promotion strategies.

## Introduction

Primary adrenal insufficiency (PAI) is characterised by inadequate steroid hormone secretion caused by intrinsic adrenal cortex disease (1). The most common causes of PAI are Addison’s disease (AD), usually resulting from autoimmune destruction of the adrenal cortex, and congenital adrenal hyperplasia (CAH), a group of autosomal recessive inborn disorders caused by enzyme deficiencies in adrenal steroidogenesis (2). PAI necessitates long-term glucocorticoid replacement, the dosage of which should be increased under stressors, such as infection, trauma or surgery, to prevent life-threatening adrenal crises, with increased stress doses recommended for patients with PAI and COVID-19 (1, 2, 3). Despite advancements in knowledge of the condition and novel treatment strategies, patients with PAI still face increased morbidity and mortality risk. Most notably, several studies have proposed that these patients may carry an increased risk of infections, which are a major trigger for adrenal crises (4, 5, 6, 7, 8, 9, 10, 11, 12).

Coronavirus disease 2019 (COVID-19) is a respiratory infection caused by the novel severe acute respiratory syndrome coronavirus 2 (SARS-CoV-2). Presentation of COVID-19 ranges from asymptomatic infection to acute respiratory distress syndrome, multiorgan failure and death. One key negative prognostic factor for severe COVID-19 is compromised immunity and increased infection risk (13); as such, patients with PAI are assumed to be particularly vulnerable to COVID-19 (9, 14). However, due to the novelty of the disease, little is known about its true impact on this patient group. A case–control study including 60 patients with PAI from an area in Italy at the epicentre of a COVID-19 outbreak reported the prevalence of symptoms suggestive of COVID-19 as similar between those with and without adrenal insufficiency, with no patients requiring hospitalisation or experiencing adrenal crises (15). A survey of 40 patients with PAI assessing the impact of the pandemic on physical and psychological health identified only one case of COVID-19, also reported as mild (16). A more recent longitudinal study exploring self-reported COVID-19-related outcomes found that patients with adrenal insufficiency perceived difficulties accessing medical care and self-managing their condition during the pandemic, resulting in feelings of anxiety and reduced quality of life (17).

Since patients with PAI may face an increased susceptibility to infections (9, 14) and there is some suggestion that patients have struggled with self-management of their disease during the pandemic, we aimed to assess the impact of COVID-19 on morbidity and health promotion attitudes of a large cohort of patients with PAI.

## Materials and methods

### Participants

Patients diagnosed with AD and CAH under the care of the Endocrinology Department at the Queen Elizabeth Hospital Birmingham and Heartlands Hospital (University Hospitals Birmingham NHS Foundation Trust, Birmingham, UK) were identified from electronic medical records. In May 2020, when the first lockdown was starting to be lifted in England, a letter containing COVID-19 information was distributed to all patients. The letter highlighted the increased risk of patients with PAI when contracting COVID-19 and potentially developing serious complications and contained advice on strict social distancing and sick day rules (Appendix Document 1, see section on supplementary materials given at the end of this article). Patients were also signposted to the latest government advice and provided with contact details for the hospital endocrinology team.

### Questionnaire and outcomes measured

A semi-structured telephone questionnaire was used to survey patients with PAI between January and April 2021 (Appendix Document 2). At this time, England had entered a third national lockdown and COVID-19 vaccinations were beginning to be rolled out, with patients prioritised for vaccination according to their age and medical history.

We gathered both demographic and clinical data; where available, clinical information was verified using electronic medical records. The Charlson Comorbidity Index was used as a measure of comorbidities to predict mortality (18). Patients were asked about their history of suspected (presence of typical symptoms (a high temperature, a new continuous cough or a loss or change to the sense of smell or taste) without polymerase chain reaction (PCR) swab confirmation) or confirmed (positive PCR swab) COVID-19 and clinical sequelae, illness within the preceding 12 months, knowledge of adrenal crisis prevention strategies as well as health promotion attitudes including ideas around the COVID-19 vaccination. PCR-positive COVID-19 was defined as severe if requiring hospital admission or triggering an adrenal crisis. An adrenal crisis was diagnosed if a patient presented with at least two of the following: hypotension or hypovolaemic shock; nausea or vomiting; severe fatigue; fever; impaired consciousness (19).

Institutional review board approval of the questionnaire and its use was obtained by the University Hospitals Birmingham National Health Service (NHS) Foundation Trust, UK (CARMS-16750).

### Statistical analysis

Categorical data were expressed as counts and percentages, and the Fisher’s exact test was applied to assess differences between groups. Quantitative variables were reported as median and interquartile range (IQR), and the Mann–Whitney *U* test was used for comparisons. Logistic regression was used to assess the odds of diagnosed or suspected COVID-19 in patients with CAH compared to those with AD. A multivariate model was used to generate an adjusted odds ratio using age, sex, body mass index (BMI), type of glucocorticoid used, daily hydrocortisone-equivalent dose, fludrocortisone treatment, Charlson Comorbidity Index score, smoking status, work and living arrangements and ethnicity as covariates. *P*-values <0.05 were considered statistically significant. The statistical analyses were carried out using GraphPad Prism 9 (GraphPad Software).

## Results

### Patient characteristics

Of 207 patients with PAI approached, 45 (AD, *N* = 29; CAH, *N* = 16) either declined to participate in the telephone interview or could not be contacted (78.3% response rate). The electronic medical records of these 45 patients were reviewed and no deaths or COVID-19-related hospital admissions were identified.

**Figure 1:**
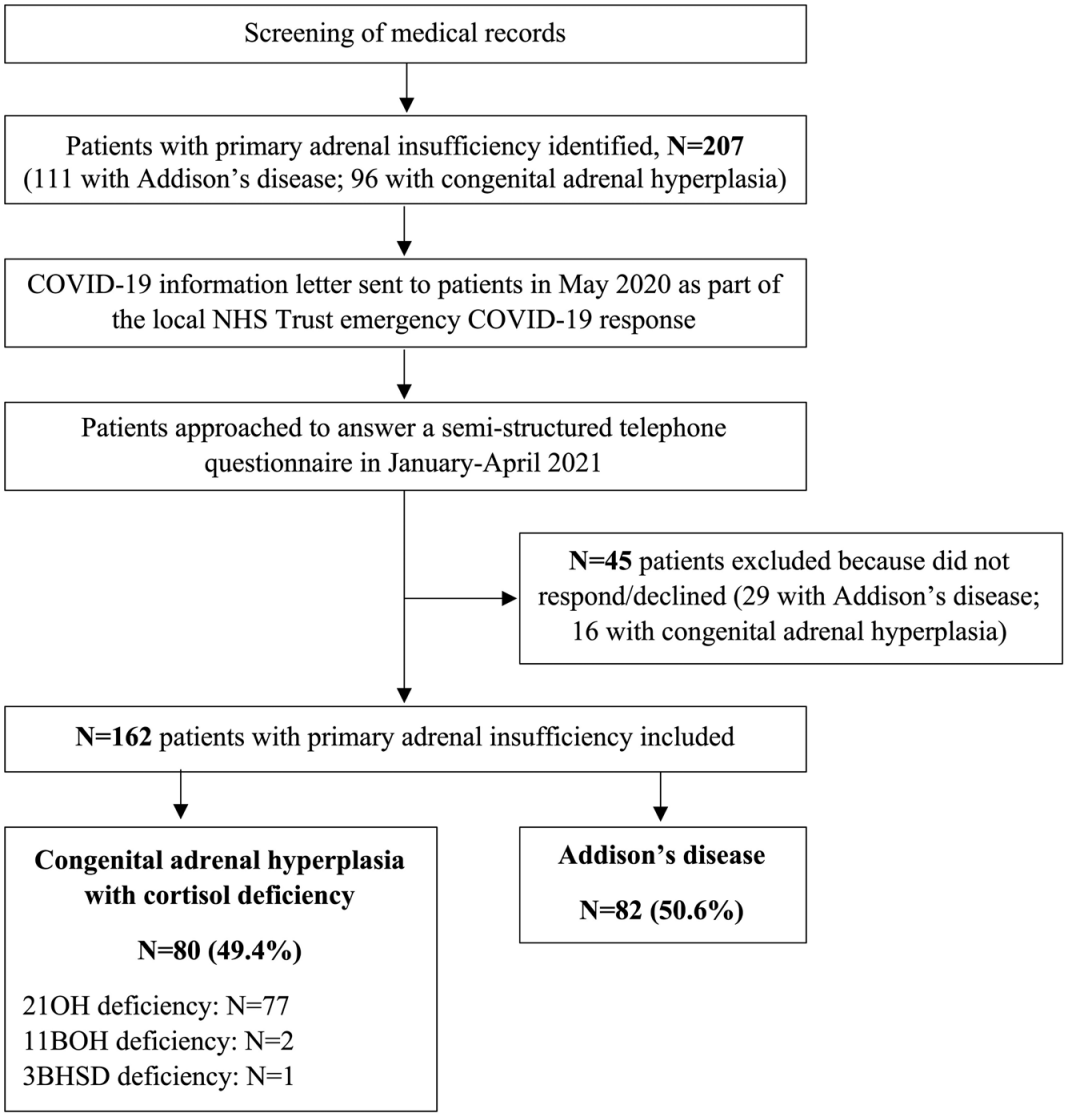
Flowchart of participant inclusion. A diagram outlining our patient recruitment process. 162 patients were included in the analysis. Patients have been categorised into their disease aetiology. Abbreviations: 21OH, 21-hydroxylase; 11BOH, 11-beta-hydroxylase; 3BHSD, 3-beta-hydroxysteroid dehydrogenase.

Of the 162 patients included in the analysis, 82 had a diagnosis of AD and 80 of CAH (Fig. 1). The most common cause of CAH was 21-hydroxylase deficiency, accounting for 96.3% of cases. Three patients with AD had a diagnosis of autoimmune polyendocrine syndrome type 1.

Most patients were women (AD, 62.2%; CAH, 64.4%). At the time of the telephone interview, the median age was 55 years (IQR 39–67) and 39 years (IQR 29–47) in patients with AD and CAH, respectively (Table 1). Both groups were similar in terms of ethnicity and BMI. Compared to patients with CAH, patients with AD were more likely to suffer from comorbid autoimmune diseases, cardiovascular disease and chronic respiratory disease (Table 1).

**Table 1:** Baseline characteristics of included patients.

	Addison’s disease (*n* = 82)	Congenital adrenal hyperplasia (*n* = 80)	*P*-value
**Women, *n* (%)**	51 (62.2)	50 (64.4)	>0.999
**Age (years), median (IQR)^a^**	55 (39–67)	39 (29–47)	<0.001
**Ethnicity, *n* (%)**			
White	73 (89.0)	62 (77.5)	0.059
Asian or Asian British	6 (7.3)	14 (17.5)	0.058
Black, African, Caribbean, or Black British	2 (2.4)	4 (5.0)	0.440
Other ethnic group	1 (1.2)	–	>0.999
**BMI (kg/m^2^), median (IQR)**	25.7 (23.2–30.0)	27.9 (22.3-31.8)	0.449
**Years since diagnosis, median (IQR)**	16 (8–28)	–	–
**Comorbidities**			
Autoimmune diseases, *n* (%)**^a^**	35 (42.7)	8 (10.0)	<0.001
Hypothyroidism, *n*	26	1	
Type 1 diabetes mellitus, *n*	12	1	
Premature ovarian failure, *n*	9	–	
Pernicious anaemia, *n*	4	1	
Hyperthyroidism, *n*	4	1	
Hypoparathyroidism*, n*	3	–	
Vitiligo,* n*	3	–	
Coeliac disease, * n*	2	1	
Alopecia, * n*	2	/	
Ulcerative colitis, * n*	1	1	
Multiple sclerosis, * n*	–	1	
Systemic lupus erythematosus, * n*	1	1	
Sarcoidosis, * n*	1	–	
Pyoderma gangrenosum, * n*	1	–	
Hypertension,* n* (%)	11 (13.4)	4 (5.0)	0.102
Asthma/COPD, * n* (%)**^a^**	11 (13.4)	3 (3.8)	0.047
Bone loss (osteopaenia/osteoporosis), * n* (%)	10 (12.2)	6 (7.5)	0.431
Reduced fertility,^b^* n* (%)	9 (11.0)	7 (8.8)	0.793
Cardiovascular events, * n* (%)^a^	8 (9.8)	1 (1.3)	0.034
Type-2 diabetes mellitus, * n* (%)	5 (6.1)	4 (5.0)	>0.999
Sleep apnoea, * n* (%)	3 (3.7)	–	0.246
Depression, * n* (%)	3 (3.7)	6 (7.5)	0.325
History of cancer, * n* (%)	2 (2.4)	1 (1.3)	>0.999
Candidiasis, * n* (%)	1 (1.2)	–	>0.999
Testicular adrenal rest tumours,* n* (% of men)	–	7 (23.3)	
Recurrent lower respiratory tract infections, * n* (%)	–	2 (2.5)	0.242
Ehlers-Danlos syndrome, * n* (%)	–	2 (2.5)	0.242
**Charlson Comorbidity Index, *n* (%)**			
0**^a^**	28 (34.1)	57 (71.3)	<0.001
1	15 (18.3)	15 (18.8)	>0.999
≥2^a^	39 (47.6)	8 (10.0)	<0.001
**Glucocorticoid replacement therapy**			
Immediate-release hydrocortisone, * n* (%)**^a^**	80 (97.6)	36 (45.0)	<0.001
Prednisolone, * n* (%)**^a^**	1 (1.2)	23 (28.8)	<0.001
Chronocort, * n* (%)	–	11 (13.8)	<0.001
Dexamethasone, * n* (%)**^a^**	–	6 (7.5)	0.013
Plenadren, * n* (%)	1 (1.2)	–	>0.999
Hydrocortisone + prednisolone, * n* (%)	–	1 (1.3)	0.494
Prednisolone + dexamethasone, * n* (%)	–	1 (1.3)	0.494
None, * n* (%)	–	2 (2.5)	0.242
Hydrocortisone-equivalent dose, median (IQR)^c^	20 (20–25)	24 (20–30)	0.328
**Mineralocorticoid replacement, * n* (%)^a^**	79 (96.3)	50 (68.5)	<0.001
Fludrocortisone dose, median (IQR)**^a^**	100 (100–188)	200 (119–200)	<0.001
**DHEA replacement, * n* (% of women)**	9 (17.6)	–	

^a^Significant figures; ^b^includes patients with premature ovarian failure; ^c^conversion factors: prednisolone 0.25; dexamethasone 0.025.

BMI, body mass index; COPD, chronic obstructive pulmonary disease; DHEA, dehydroepiandrosterone; IQR, interquartile range.

About 97.6% of patients with AD were treated with immediate-release hydrocortisone, compared to 45.0% of patients with CAH. Other steroid replacement therapies in patients with CAH included prednisolone (28.8%), modified-release hydrocortisone (13.8%) and dexamethasone (7.5%). The hydrocortisone-equivalent dosages did not differ significantly between the two groups. About 96.3% of patients with AD and 68.5% of patients with CAH were treated with mineralocorticoid replacement therapy, with the latter group receiving a higher daily dose of fludrocortisone (Table 1).

### Illness during the COVID-19 pandemic

**Figure 2:**
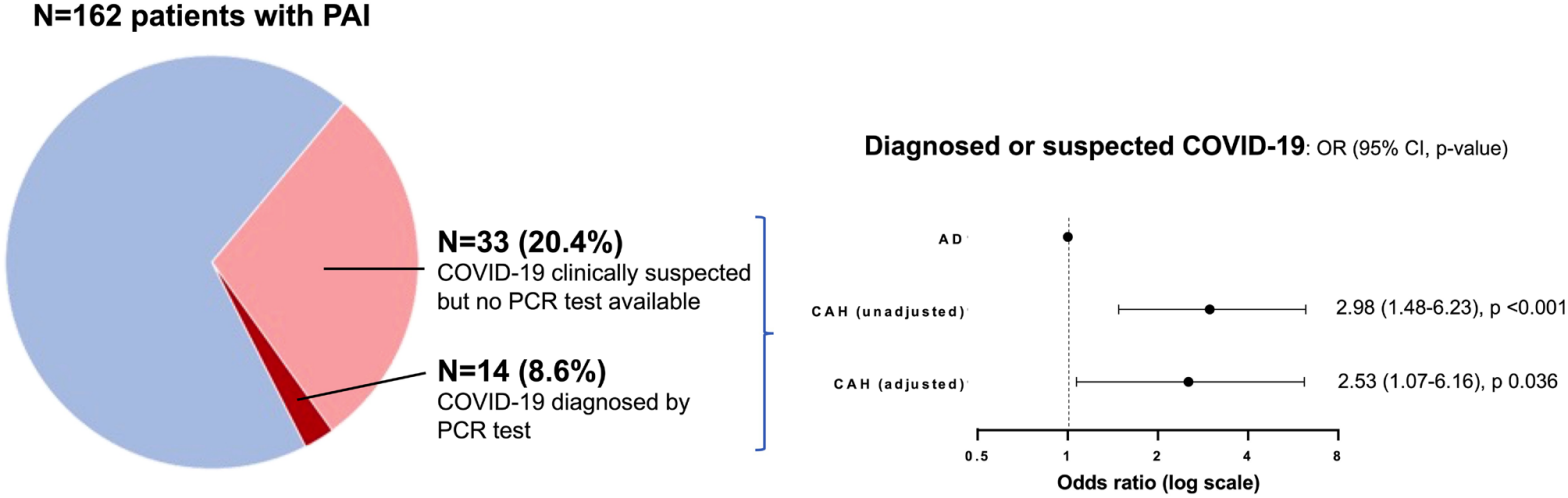
Prevalence of COVID-19 in the study participants. Forty-seven patients with primary adrenal insufficiency (PAI) had suspected or confirmed COVID-19 based on symptoms or a positive polymerase chain reaction (PCR) test. Patients with congenital adrenal hyperplasia (CAH) had significantly higher odds of COVID-19 compared to those with Addison’s disease (AD).

Forty-seven patients (29.0%) were suspected to have had COVID-19 by the time the questionnaire was completed (Table 2 and Fig. 2). Only a minority were tested, and the infection was confirmed by PCR test in five patients with AD and nine with CAH. Severe COVID-19 affected two patients with AD and two with CAH (Appendix Table 1). No deaths were recorded.

**Table 2:** COVID-19 infection status and health during the preceding 12 months.

	Addison’s disease (*n* = 82)	Congenital adrenal hyperplasia (*n* = 80)	*P*-value
**COVID-19 diagnosed or suspected, *n* (%)**^a^	15 (18.3)^b^	32 (40.0)	0.003
COVID-19 diagnosed	5	9	
Severe disease	2	2	
COVID-19 suspected but subject not tested	10	23	
**Had sick days in the past 12 months, *n* (%)**	51 (62.2)^c^	50 (62.5)^c^	>0.999
Malaise/fatigue	17	15	
COVID-19 (confirmed or suspected)	10	15	
Gastroenteritis	8	8	
Urinary tract infection	4	3	
Upper respiratory tract infection	3	10	
Lower respiratory tract infection	3	1	
Skin infection	3	–	
Tooth abscess	3	–	
Flu-like symptoms	3	5	
Psychological stress/bereavement	3	2	
Physical trauma	2	1	
COVID-19 vaccine/flu jab	2	2	
Ear infection	2	1	
Irritable bowel syndrome	–	3	
Physical activity	–	2	
Headache/migraine	–	2	
Cough	–	1	
Non-specific symptoms	–	1	
**Had an adrenal crisis in the past 12 months, *n* (%)**	10 (12.2)	8 (10.0)^d^	0.804
COVID-19 (confirmed or suspected)	2	2	
Gastroenteritis	2	1	
Urinary tract infection	2	–	
Dental abscess	1	–	
Flu-like symptoms	1	–	
Zoledronate infusion	1	–	
Unknown trigger	1	5	
Physical activity	/	1	
**Had hospital admissions in the past 12 months, *n* (%)^e^**	8 (9.8)	14 (17.5)^d^	0.174
Physical trauma/traumatic fracture	2	2	
Lower respiratory tract infection	–	3	
Gastroenteritis	1	1	
Surgery (planned or emergency)	1	2	
Transient ischaemic attack	–	2	
Urinary tract infection	–	2	
Chest pain	1	1	
Hypertension, tachycardia	1	–	
Miscarriage	1	–	
Delivery	–	1	
Diabetic ketoacidosis	–	1	
Cellulitis	1	–	
Migraine	–	1	

^a^Significant figure; ^b^of the three patients with autoimmune polyendocrine syndrome type 1, one developed mild symptoms of COVID-19 and did not require hospital admission; ^c^some patients had multiple sick days; ^d^one patient had two adrenal crises and three hospital admissions; ^e^excluding hospital admissions for adrenal crisis and/or severe COVID-19.

Patients with CAH were more likely to have had a diagnosis of confirmed or clinically suspected COVID-19 (40.0% vs 18.3% of patients with AD; unadjusted odds ratio 2.98 (95% CI 1.48–6.23), *P* < 0.001). This remained significant after adjustment for age, sex, BMI, type of glucocorticoid used, daily hydrocortisone-equivalent dose, fludrocortisone treatment, Charlson Comorbidity Index score, smoking status, employment, living arrangements and ethnicity (adjusted odds ratio 2.53 (95% CI 1.07–6.16), *P* = 0.036). In line with the higher odds observed in patients with CAH, the 47 patients with PAI and suspected or confirmed COVID-19 were less commonly treated with immediate-release hydrocortisone or fludrocortisone and had a lower burden of comorbidity (Appendix Table 2). Subgroup analyses in patients with AD showed that those with previously suspected or confirmed COVID-19 were younger, had a lower cardiometabolic burden and were more likely to work at the time of the interview (Appendix Table 3). Patients with CAH and previously suspected or confirmed COVID-19 were older and less likely to be treated with mineralocorticoid-replacement therapy (Appendix Table 4).

**Table 3:** COVID-19 risk factors and health promotion attitudes.

	Addison’s disease (*n* = 82)	Congenital adrenal hyperplasia (*n* = 80)	*P*-value
**Smoking habit, *n* (%)**			
Non-smoker	65 (79.3)	70 (87.5)	0.206
Former smoker	8 (9.8)	6 (7.5)	0.781
Current smoker	9 (11.0)	4 (5.0)	0.247
**Currently working, *n* (% of currently working)**	34 (41.5)	35 (43.8)	0.874
Key workers^ a^	10 (29.4)	18 (51.4)	0.087
Able to work from home	17 (50.0)	16 (45.7)	0.811
**Household arrangement, *n* (%)**			
Lives alone	14 (17.1)	9 (11.3)	0.369
Lives with another person	35 (42.7)	31 (38.8)	0.635
Lives with more than one person	33 (40.2)	33 (41.3)	>0.999
**Has the NHS COVID-19 app, *n* (%)**	33 (40.2)	41 (51.3)	0.207
**Had/planning to have the COVID-19 vaccine, *n* (%)**^b^	79 (96.3)	64 (80.0)	0.001
**Had/planning to have the flu vaccine this year, *n* (%)**^b^	62 (75.6)	41 (51.3)	0.002
**Has the flu vaccine every year, *n* (%)**^b^	60 (73.2)	42 (52.5)	0.009
**Is aware of the sick day rules, *n* (%)**^b^	82 (100)	75 (93.8)	0.028
**Has a steroid emergency card, *n* (%)**	75 (91.5)	70 (87.5)	0.452
Always carries the card	63 (84.0)	62 (88.6)	0.477
Carries the card just occasionally	10 (13.3)	5 (7.1)	0.280
Never carries the card	2 (2.7)	5 (7.1)	0.263
**Has a steroid injection kit, *n* (%)**	76 (92.7)	70 (87.5)	0.303
It is up to date	54 (71.1)	43 (61.4)	0.226
It is out of date	19 (25.0)	22 (31.3)	0.462
Does not know whether it is still in date	3 (3.9)	5 (7.1)	0.481
**Has received training for self-injection, *n* (%)**^b^	75 (91.5)	64 (80.0)	0.044
**Family members have received injection training, *n* (%)**	54 (65.9)	54 (67.5)	0.869
**Is confident in self-injecting,* n* (%)**	54 (65.9)	43 (53.8)	0.149
**Has ever self-injected, *n* (%)**	17 (20.7)	17 (21.3)	>0.999
**Wears medical alert jewellery, n (%)** ^b^	53 (64.6)	29 (36.3)	0.001

^a^Occupation groups: health and social care, education and childcare, food and other necessary goods; key public services; local and national government; utilities and communication; public safety and national security; transport; ^b^significant figures. NHS, National Health Service.

Within the preceding 12 months of interview, 62.3% of patients with PAI had to follow sick day rule advice for intercurrent illness, with suspected or confirmed COVID-19 being the second most common reason after general malaise/fatigue (18.5% and 23.7% of all sick day events, respectively). During the year preceding the interview, 18 patients with PAI (11.1%) suffered at least one adrenal crisis and suspected or confirmed COVID-19 was the leading trigger where a trigger could be identified, with four cases reported (Table 2).

### COVID-19 risk factors and health promotion attitudes

With regards to social risk factors for developing COVID-19 enquired about in the interviews (smoking status, employment and living arrangements), there was no significant difference between patients with AD or CAH (Table 3).

More patients with AD had received, or planned to receive, the COVID-19 vaccine compared to patients with CAH (96.3% and 80.0%, respectively; *P* < 0.001). More patients with AD had also received, or planned to receive, the flu vaccine during the 2020–21 period (75.6% vs 51.3%; *P* < 0.002) and reported receiving it every year (73.2% vs 52.5%; *P* < 0.009). All patients with AD were aware of sick day rules, compared to 93.8% of patients with CAH. Most patients with PAI had a steroid emergency card (89.5%) and a hydrocortisone self-injection kit available (90.1%) at the time of the interview, but patients with AD were more likely to have received self-injection training (91.5% vs 80.0% of patients with CAH;*P* < 0.044). Medical alert jewellery was worn more frequently by patients with AD (64.6% vs 36.3%; 0.001). Less than half of the patients included in the study reported having the National Health Service COVID-19 application on their phones (AD, 40.2%; CAH, 51.3%) (Table 3).

## Discussion

In this cross-sectional study, we demonstrated that COVID-19 has been a major precipitant for ill health in a large cohort of patients with PAI, commonly leading to sick day events and even culminating in hospital admission for adrenal crisis in four patients. We found higher odds of suspected or confirmed COVID-19 among patients with CAH compared to those with AD.

Infections, and most importantly those of the respiratory system, are an established cause of poor health outcomes in patients with PAI, resulting in increased morbidity and mortality (5, 7, 8, 9). Studies have found a higher incidence of infections, antimicrobial agent prescribing, and hospital admissions for infections compared to matched controls (9, 20), and patients are also more likely to develop serious clinical consequences; even infections that may cause mild illness amongst the immunocompetent population can result in adrenal crisis and death (5, 7, 8, 21). One explanation for the possible increased susceptibility to infection in patients with AD is the impaired neutrophil and natural killer cell function, compromising recognition and elimination of infected cells by the innate immune system (14). Despite best efforts, currently available steroid preparations do not accurately replicate physiological glucocorticoid responses, potentially resulting in adverse changes to immune function in patients with PAI (9, 14). Furthermore, the role of serum cortisol in priming the immune system means patients with PAI may be unable to mount a sufficient acute response to infection, thus increasing the likelihood of complications (22, 23). Another mechanism that has been proposed could be dehydroepiandrosterone (DHEA) deficiency in patients with AD, since DHEA may play a role in regulating immune function (9, 24). Only 9 women with AD in our study were treated with DHEA replacement; hence, we could not carry out a subgroup analysis of the impact of this treatment on COVID-19 risk.

We found that 4 out of 47 patients with suspected or confirmed COVID-19 (8.5%) were admitted to the hospital for severe disease. Our results are in line with a recently published study by Öster S *et al.* (25); in a large Swedish cohort of patients with AD, the authors found that 17% of patients reported a diagnosis of COVID-19, of which 8.5% required hospital admission (25). An analysis of the European Registries for Rare Endocrine Conditions found a higher risk of hospital admission (14%) in a group of 57 patients with adrenal insufficiency (26).

Several agencies have released guidelines to advise both clinicians and patients as to the risks associated with COVID-19 in PAI (3, 23, 27, 28, 29). Advice has primarily relied on expert opinion, owing to the rarity of original clinical data. Due to concerns over a higher risk of complications and mortality, the European Society of Endocrinology advises that sick day rules, where patients at least double steroid replacement therapy during periods of physiological stress, should be implemented immediately in suspected or confirmed COVID-19, even if symptoms are only mild. Patients are also recommended to ensure they have adequate supplies of replacement therapy at home, in case of further national lockdowns or the requirement for self-isolation (29). Other guidelines emphasise the importance of empowering patients to take a proactive stance towards their own health promotion during the pandemic, through means such as education around social distancing and discussion of current COVID-19 guidelines (3).

Our data highlight differences in COVID-19 rates, and attitudes towards illness prevention, between patients with AD and CAH. The latter were more likely to develop suspected or confirmed COVID-19 during the study period, which is in line with a recent study by Yedinak C and Ross IL (30). The authors conducted a multinational survey on patients with a self-reported diagnosis of adrenal insufficiency and found that 8 of 72 patients with CAH (11.1%) tested positive for COVID-19 as compared to 32 of 1219 patients with other adrenal insufficiency aetiologies (2.6%) (30). The authors also found that COVID-19-positive patients were treated with higher daily hydrocortisone replacement doses, whilst we did not observe this.

While patients with CAH experienced higher rates of suspected or confirmed COVID-19 in our study, they also demonstrated reduced self-protective attitudes and engagement with healthcare services. For example, 80% of patients with CAH had received, or planned on receiving, the COVID-19 vaccine at the time of the interview, compared to almost all patients with AD. With regards to the flu vaccine, only about half of patients with CAH reported receiving it every year, compared to around three-quarters of those with AD. Additionally, patients with CAH were significantly less likely to have received training in self-injection or to wear medical alert jewellery. Prior work has identified problems in transitioning patients with CAH from paediatric to adult endocrine services, with patients often being lost to follow-up (31, 32, 33). In 2010, it was found that less than 10% of the expected UK adult population of patients with CAH were under specialist endocrine care (32). Considering this, the reduced self-protective attitudes observed in this study are perhaps unsurprising. It is imperative to ensure that patients with PAI, especially those with CAH, are having regular follow-up appointments with some focus on health promotion education and reinforcement of self-management skills.

Besides reduced engagement with healthcare services, our results highlight other areas that may contribute to increased rates of COVID-19 among patients with CAH. Patients with AD were older and had a greater burden of comorbidity than those with CAH. It may be that patients with AD perceived themselves to be at a greater risk of falling unwell with COVID-19 for these reasons and therefore felt more inclined to follow our shielding advice stringently. It is also worth appreciating our data on employment. Although a similar number of patients with CAH and AD were working at the time of the interview, 51.4% of employed patients with CAH were key workers who continued to work as normal during the pandemic, compared to only 29.4% of those with AD. These patients likely faced higher levels of exposure to COVID-19.

This study benefits from its large cohort of well-characterised patients with PAI, with a sample size large enough to enable comparisons between CAH and AD. Additionally, the use of a semi-structured questionnaire allowed the collection of comprehensive qualitative data and a thorough exploration of patients’ health attitudes. However, the present study also has some limitations. First, we relied on patient-reported suspicion of COVID-19 for many cases, as PCR testing in the UK was not available out-of-hospital to the wider public until mid-2020 (34). A patient’s interpretation of symptoms could be inaccurate, resulting in discrepancies between suspected and confirmed cases of COVID-19. Second, we did not perform a case–control or population-based analysis with matched subjects without PAI. This would have helped in determining whether patients with PAI truly carried a higher risk of COVID-19 compared to people with normal adrenal function. Finally, the single-centre nature of this study limits the generalisability of our findings.

In conclusion, we found that patients with CAH had greater odds of contracting COVID-19 than patients with AD and reduced self-protective healthcare attitudes have likely contributed to this. Patients with PAI must understand their heightened susceptibility to infection and the importance of self-protective attitudes to prevent health deterioration.

## Supplementary Materials

Supplementary Material

## Conflict of Interest

The authors do not declare a conflict of interest in relation to this work.

## Funding

L.M.S. is the recipient of a Health Education England/National Institute for Health and Care Research http://dx.doi.org/10.13039/501100000272 (HEE/NIHR) doctoral fellowship (grant reference number ICA CDRF-2018-04-ST2-050). A.P. is the recipient of a Diabetes UK http://dx.doi.org/10.13039/501100000361 Sir George Alberti Research Training (grant reference number 18/0005782). A.P. receives support from the NIHR Birmingham Biomedical Research Centre http://dx.doi.org/10.13039/501100018952 at the University Hospitals http://dx.doi.org/10.13039/100012324 Birmingham NHS Foundation Trust and the University of Birmingham (grant reference number NIHR203326). The views expressed are those of the author(s) and not necessarily those of the NIHR or the Department of Health and Social Care http://dx.doi.org/10.13039/501100000276 UK.

## Data availability

De-identified, individual participant-level data are available upon reasonable request. All requests should be sent to the corresponding author.
