# Supplementary material for: Impact of coronavirus disease 2019 on patients with primary adrenal insufficiency: a cross-sectional study
Source: Endocr Connect. 2023 Jul 12;12(8):e230122. doi: 10.1530/EC-23-0122 (PMC10388655; doi:10.1530/EC-23-0122)
Supplement: Supplementary Material [file supplementary_material.pdf]

Impact of COVID-19 on patients with primary adrenal insufficiency: a cross-sectional study

APPENDIX

Appendix document 1. COVID-19 information letter sent to patients with PAI in May 2020.

University Hospitals Birmingham NHS
NHS Foundation Trust

IMPORTANT INFORMATION ON COVID-19 FOR PATIENTS WITH ADRENAL INSUFFICIENCY

Due to your adrenal insufficiency, you are classed as at **increased risk** of both contracting coronavirus and developing serious complications from the infection. You need to follow the Government's advice on **stringent social distancing, staying at home, working from home if possible and regular handwashing**. Details of the Government's most up-to-date advice can be found on the link below.
https://www.gov.uk/government/publications/covid-19-guidance-on-social-distancing-and-for-vulnerable-people/guidance-on-social-distancing-for-everyone-in-the-uk-and-protecting-older-people-and-vulnerable-adults

The Government has also published a guideline for people who are classed as **extremely vulnerable** and would need 12-week shielding. Most patients in England under this category have already been notified. If you have not received any notification from the NHS, this means that you are **not** classed as extremely vulnerable but rather as increased risk. Details of this guideline on shielding can be found on the link below.
https://www.gov.uk/government/publications/guidance-on-shielding-and-protecting-extremely-vulnerable-persons-from-covid-19/guidance-on-shielding-and-protecting-extremely-vulnerable-persons-from-covid-19

Please find below key information from the Society for Endocrinology for patients with Adrenal Insufficiency (please read them carefully).

Please do not hesitate to contact us for any queries by email (preferred method) or by phone on

University Hospitals Birmingham NHS
NHS Foundation Trust

Society for Endocrinology

Advice for patients who take replacement steroids (hydrocortisone, prednisolone, dexamethasone or plenadren) for pituitary/adrenal insufficiency

If you are on steroid replacement therapy for pituitary or adrenal disease, or care for someone who is, and you're worried about Coronavirus, we've brought together a number of resources that we hope you will find useful.

The World Health Organization has declared the Coronavirus outbreak to be a public health emergency of international concern. In response, the UK Chief Medical Officers have raised the risk to the public.

If you have adrenal insufficiency (steroid dependent) and you become unwell for any reason, it's important that you follow sick day rules. This means in the first instance that you double your usual doses of glucocorticoid replacement.

The term adrenal insufficiency includes Addison's disease with primary adrenal failure, patients with pituitary problem including secondary adrenal failure and patients with congenital adrenal hyperplasia.

The government has recently recommended that those at increased risk of severe illness from coronavirus should be particularly stringent in following social distancing measures. On the basis of current data, there is no evidence that patients with adrenal insufficiency are at increased risk of contracting COVID-19. However, we know that patients with Addison's disease (primary adrenal insufficiency) and congenital adrenal hyperplasia have a slightly increased overall risk of catching infections. In addition, patients with adrenal insufficiency would require additional measures when severely unwell (hydrocortisone injection and infusion) to avoid an adrenal crisis.

The recently published list of people considered vulnerable includes anyone instructed to get a flu jab as an adult each year on medical grounds as well as all people whose medication includes steroid tablets. Whilst adrenal insufficiency is not specifically mentioned in this guidance, both of these criteria apply to patients with adrenal insufficiency. Patients with adrenal insufficiency are recommended to have flu vaccinations, as this would not only prevent illness from the flu itself but also reduce complications arising from managing the adrenal insufficiency during severe illness. We would therefore consider patients with adrenal insufficiency part of the group of vulnerable individuals who should, therefore, be particular stringent in following social distancing measures.

The government and NHS are continually updating their advice and information in relation to Coronavirus. These resources are the most up-to-date information currently available, so we recommend you check them first if you have any worries or concerns.

Where to get general information:

The most up-to-date information about the outbreak, including information for returning travellers, can be found on the gov.uk website and at https://www.nhs.uk/conditions/coronavirus-covid-19/

Tailored information for people living in Wales, Scotland and Northern Ireland can also be found at:

- Public Health Wales
- Health Protection Scotland
- Public Health Agency (NI)

The NHS also has up-to-date advice on what to do if you've recently returned from travel abroad, symptoms to look out for, and action to take if you become unwell.
The Foreign and Commonwealth Office has information and advice for British people travelling and living overseas.

Specific information for patients with adrenal insufficiency:

- At present we do not know if patients with adrenal insufficiency are at higher risk of catching coronavirus' and we advise patients with adrenal insufficiency to follow recommendations on the NHS website https://www.nhs.uk/conditions/coronavirus-covid-19/ to minimise risk of catching and transmitting the virus. All patients with symptoms suggestive of COVID-19 or patients who are unwell with confirmed COVID-19 should follow the usual steroid sick day rules:

**Sick Day Rule 1**:<br>a. Double you daily oral steroid dose.<br>b. If you have a fever, take paracetamol 1000mg every 6 hours.<br>c. If your fever persists, **increase your hydrocortisone dose further to 20mg every 6 hours**.<br>d. Keep well hydrated by drinking regularly throughout the day, even during the night.
**Sick Day Rule 2**:<br>a. Administer glucocorticoids (via intramuscular injection [or intravenous injection in hospital setting]) during prolonged vomiting or diarrhoea, during preparation for colonoscopy or in case of acute trauma or surgery.<br>b. Seek medical help if your condition worsens.

If in doubt, please contact your endocrine team for advice as detailed below.

- Patients should make sure they have sufficient additional hydrocortisone supply including injections.
- Patients should make sure the doctors and other health care professionals looking after them are aware that they require an increased dose of steroids or steroids as injection as per sick day rules.

If you become unwell, in non-emergency situations, NHS 111 will be able to give specific advice. In an emergency, call 999 immediately.

**Contact Details<br>University Hospitals Birmingham NHS Foundation Trust- Endocrinology Department**	
Queen Elizabeth Hospital Birmingham	Email (preferred method): *Redacted*<br>Phone: *Redacted*<br>(we will aim to return telephone call within 3-5 working days)
Heartlands, Solihull and Good Hope Hospitals	Email (preferred method): *Redacted*<br>Phone: *Redacted*<br>(we will aim to return telephone call within 3-5 working days)

Appendix Document 2. Survey conducted over telephone interviews.

Clinical Information and Demographics

- Are you a smoker?
  - Yes
  - Former smoker
  - No
- What is your most recent height/weight?
- Do you work?
  - Yes (record details – key workers, working from home, etc.)
  - No
- What is your ethnicity?
  - White
  - Mixed or Multiple ethnic groups
  - Asian or Asian British
  - Black, African, Caribbean or Black British
  - Other ethnic group
- What are your household arrangements?
  - Live alone
  - Live with another person (e.g. partner, son, friend, etc.)
  - Live with more than one person
  - Other [open field]

COVID-19 Information

- Have you been diagnosed with COVID-19? If yes, collect information about:
  - what kind of symptoms
  - date of onset of symptoms
  - date of diagnosis
  - duration of symptoms
  - need for sick day steroids
  - need for / duration of hospital admission
- If you have not been formally diagnosed, have you been suspected to have COVID-19 (the main symptoms are: a high temperature; a new, continuous cough; a loss or change to your sense of smell or taste)? If yes, collect information about:
  - what kind of symptoms
  - date of onset of symptoms
  - duration of symptoms
  - need for sick day steroids
  - need for / duration of hospital admission
- Do you have the NHS COVID-19 app downloaded to your phone?
  - Yes
  - No
- Did you have (or planning to have) the COVID-19 vaccine?
  - Yes
  - No
- Did you have (or planning to have) the flu jab this year?
  - Yes
  - No
- Do you have the flu jab every year?
  - Yes
  - No

Prevention strategies & Adrenal crisis management

- Do you have a steroid card?
  - Yes
  - No
- Do you carry your steroid card with you all the time?
  - Yes, always

    - No, just occasionally
    - No, never
    - I don't have a steroid card
    - Other [open field]
- Do you wear medical alert jewellery stating that you are steroid dependent?
    - Yes
    - No
- Are you aware of the sick day rules?
    - Yes
    - No
- Do you have an emergency hydrocortisone injection kit?
    - No
    - Yes, and it is up to date (issued in the last 12 months)
    - Yes, but it is out of date (older than 12 months)
    - Yes, but I don't remember when it was issued last time
- Have you been trained in using the emergency hydrocortisone injection kit?
    - Yes – face-to-face training or online via video?
    - No
- Has a member of your family or another person close to you been trained in using the emergency hydrocortisone injection kit?
    - Yes
    - No
- Do you feel confident using the emergency hydrocortisone injection kit?
    - Yes
    - No
- Have you ever self-injected hydrocortisone (emergency hydrocortisone injection kit)?
    - Yes
    - No
- Did you have to double your usual steroid dose during the last 12 months?
    - Yes (ask for details – how many times, triggering factors, duration of the event, etc.)
    - No
- Have you needed emergency treatment for an adrenal crisis during the last 12 months? (hydrocortisone injection and/or IV fluids via a drip)
    - Yes (ask for details – how many times, triggering factors, duration of the event, etc)
    - No
- Did you have any hospital admissions during the last 12 months?
    - Yes – Follow-up question: what was the reason for admission? how was your experience? (open question: management of adrenal insufficiency by hospital staff, etc.)
    - No

Appendix table 1. Clinical characteristics and clinical course of the patients who developed severe COVID-19. Abbreviations: 21-OHD, 21-hydroxylase deficiency; AD, Addison's disease; BMI, body mass index; CAH, congenital adrenal hyperplasia; CPAP, continuous positive airway pressure; DHEA, dehydroepiandrosterone; HDU, high dependency unit; T1DM, type 1 diabetes mellitus; T2DM, type 2 diabetes mellitus.

	**Diagnosis**	**Demographics**	**Comorbidities**	**Adrenal insufficiency treatment**	**Hospital admission details**
**Case 1**	21-OHD<br>CAH	Woman<br>49 years<br>White<br>BMI 31.5 kg/m^2	T2DM<br>Asthma<br>Depression	Chronocort 20 mg daily<br>Fludrocortisone 250 µg daily	Patient admitted with asthma exacerbation.<br>10-day admission.
**Case 2**	21-OHD<br>CAH	Man<br>41 years<br>Asian<br>BMI 33.3 kg/m^2	Osteoarthritis	Hydrocortisone 20 mg daily<br>Fludrocortisone 150 µg	Patient admitted with adrenal crisis.<br>Required CPAP on HDU.<br>13-day admission.
**Case 3**	AD	Woman<br>54 years<br>White<br>BMI 34.2 kg/m^2	Asthma	Hydrocortisone 20 mg daily<br>Fludrocortisone 150 µg daily<br>DHEA 50 mg daily	Patient admitted with asthma exacerbation.<br>5-day admission.
**Case 4**	AD	Woman<br>31 years<br>White<br>BMI 20.3 kg/m^2	T1DM	Hydrocortisone 30 mg daily<br>Fludrocortisone 200 µg	Patient admitted with adrenal crisis.<br>3-day admission.

Appendix table 2: Clinical characteristics of patients with primary adrenal insufficiency with or without COVID-19 diagnosed or suspected.

	COVID-19 diagnosed or suspected (n=47)	No COVID-19 (n=115)	p-value
**Aetiology of primary adrenal insufficiency**			
Addison's disease, n (%)[*]	15 (31.9)	67 (58.3)	0.003
Congenital adrenal hyperplasia, n (%)	32 (68.1)	48 (41.7)	
**Women, n (%)**	32 (68.1)	69 (60.0)	0.375
**Age (years), median (IQR)**	41 (35-53)	48 (33-61)	0.158
**Ethnicity**			
White, n (%)	40 (85.1)	95 (82.6)	0.818
Asian or Asian British, n (%)	5 (10.6)	15 (13.0)	0.796
Black, African, Caribbean, or Black British, n (%)	2 (4.3)	4 (3.5)	>0.999
Other ethnic group, n (%)	/	1 (0.9)	>0.999
**BMI (kg/m^2), median (IQR)**	25.6 (20.5-31.0)	26.8 (23.6-30.3)	0.299
**Glucocorticoid replacement therapy**			
Immediate-release hydrocortisone, n (%)[*]	28 (59.6)	90 (78.3)	0.020
Prednisolone, n (%)	10 (21.3)	12 (10.4)	0.080
Chronocort, n (%)	6 (12.8)	5 (4.3)	0.081
Dexamethasone, n (%)	2 (4.3)	4 (3.5)	>0.999
Plenadren, n (%)	1 (2.1)	/	0.290
Hydrocortisone + prednisolone, n (%)	/	1 (0.9)	>0.999
Prednisolone + dexamethasone, n (%)	/	1 (0.9)	>0.999
None, n (%)	/	2 (1.7)	>0.999
Hydrocortisone-equivalent dose, median (IQR)[a]	25 (20-30)	20 (20-25)	0.222
**Mineralocorticoid replacement, n (%)[*]**	32 (68.1)	103 (89.6)	0.002
Fludrocortisone dose, median (IQR)	200 (100-200)	150 (100-200)	0.263
**DHEA replacement, n (% of women with Addison's)**	2 (20.0)	7 (17.1)	>0.999
**Comorbidities**			
Autoimmune diseases, n (%)	10 (21.3)	30 (26.1)	0.555
Type 2 diabetes, n (%)	2 (4.3)	7 (6.1)	>0.999
Hypertension, n (%)	3 (6.4)	1 (0.9)	0.074
Cardiovascular events, n (%)	1 (2.1)	6 (5.2)	0.674
Asthma / COPD, n (%)	4 (8.5)	10 (8.7)	>0.999
Recurrent lower respiratory tract infections, n (%)	2 (4.3)	/	0.083
Sleep apnoea, n (%)	/	3 (2.6)	0.557
Depression, n (%)	2 (4.3)	7 (6.1)	>0.999
Bone loss (osteopaenia/osteoporosis), n (%)	3 (6.6)	13 (11.3)	0.402
Ehlers-Danlos syndrome, n (%)	1 (2.71)	1 (0.9)	0.497
Testicular adrenal rest tumours, n (% of men with CAH)	2 (22.2)	5 (25.0)	>0.999
History of cancer, n (%)	/	3 (2.6)	0.557
Reduced fertility[b], n (%)	4 (8.5)	8 (7.0)	0.746
Candidiasis, n (%)	/	1 (0.9)	>0.999
**Charlson Comorbidity Index**			
0, n (%)	30 (63.8)	56 (48.7)	0.086
1, n (%)	9 (19.1)	20 (17.4)	0.823
≥2, n (%)[*]	8 (17.0)	39 (33.9)	0.036
**Smoking habit**			
Non-smoker, n (%)	38 (80.9)	97 (84.3)	0.644
Former smoker, n (%)	5 (10.6)	9 (7.8)	0.550
Current smoker, n (%)	4 (8.5)	9 (7.8)	>0.999
**Currently working, n (%)**	25 (53.2)	45 (39.1)	0.117
Key workers, n (% of currently working)	12 (48.0)	19 (42.2)	0.802
Able to work from home, n (% of currently working)	9 (36.0)	25 (55.6)	0.140
**Household arrangement**			
Lives alone, n (%)	7 (14.9)	17 (14.8)	>0.999
Lives with another person, n (%)	16 (34.0)	53 (46.1)	0.167

Lives with more than one person, n (%)	24 (51.1)	45 (39.1)	0.220
**Has the NHS COVID-19 app, n (%)**	25 (53.2)	49 (42.6)	0.230
**Had/planning to have the COVID-19 vaccine, n (%)**	44 (93.6)	99 (86.1)	0.281
**Had/planning to have the flu vaccine this year, n (%)**	33 (70.2)	70 (60.9)	0.286
**Has the flu vaccine every year, n (%)**	34 (72.3)	68 (59.1)	0.151
**Is aware of the sick day rules, n (%)**	46 (97.9)	111 (96.5)	>0.999
**Has a steroid emergency card, n (%)**	42 (89.4)	103 (89.6)	>0.999
Always carries the card, n (%)	38 (90.5)	87 (84.5)	0.433
Carries the card just occasionally, n (%)	1 (2.4)	14 (13.6)	0.066
Never carries the card, n (%)	3 (7.1)	4 (3.9)	0.413
**Has a steroid injection kit, n (%)**	42 (89.4)	104 (90.4)	0.780
It is up to date, n (%)	28 (66.7)	69 (66.3)	>0.999
It is out of date, n (%)	12 (28.6)	29 (27.9)	>0.999
Does not know whether it is still in date, n (%)	2 (4.8)	6 (5.8)	>0.999
**Has received training for self-injection, n (%)**	43 (91.5)	96 (83.5)	0.223
**Family members have received injection training, n (%)**	32 (68.1)	76 (66.1)	0.856
**Is confident in self-injecting, n (%)**	26 (55.3)	71 (61.7)	0.483
**Has ever self-injected, n (%)**[*]	17 (36.2)	17 (14.8)	0.005
**Wears medical alert jewellery, n (%)**	22 (46.8)	60 (52.2)	0.605
**Had sick days in the past 12 months, n (%)**[c]	27 (57.4)	62 (53.9)	0.730
**Had an adrenal crisis in the past 12 months, n (%)**	7 (14.9)	11 (9.6)	0.409
**Had hospital admissions in the past 12 months, n (%)**[d]	9 (19.1)	13 (11.3)	0.210

Abbreviations: COVID-19, Coronavirus disease 2019; IQR, interquartile range; BMI, body mass index; DHEA, Dehydroepiandrosterone; COPD, chronic obstructive pulmonary disease; NHS, National Health Service.

[a] Conversion factors: prednisolone 0.25; dexamethasone 0.025.

[b] Includes patients with premature ovarian failure.

[c] Excluding sick days because of confirmed or suspected COVID-19.

[d] Excluding hospital admissions for adrenal crisis and/or severe COVID-19.

[*] Significant figures.

Appendix table 3: Clinical characteristics of patients with Addison's disease with or without COVID-19 diagnosed or suspected.

	COVID-19 diagnosed or suspected (n=15)	No COVID-19 (n=67)	p-value
**Women, n (%)**	10 (66.7)	41 (61.2)	0.775
**Age (years), median (IQR)***	41 (32-52)	56 (47-71)	0.003
**Ethnicity**			
White, n (%)	15 (100)	58 (86.6)	0.200
Asian or Asian British, n (%)	/	6 (9.0)	0.586
Black, African, Caribbean, or Black British, n (%)	/	2 (3.0)	>0.999
Other ethnic group, n (%)	/	1 (1.5)	>0.999
**BMI (kg/m^2), median (IQR)**	25.6 (21.9-27.5)	26.1 (23.6-30)	0.506
**Glucocorticoid replacement therapy**			
Hydrocortisone alone – any formulation, n (%)	14 (93.3)	66 (98.5)	0.334
Any other treatment, n (%)	1 (6.7)	1 (1.5)	
Hydrocortisone-equivalent dose, median (IQR)[a]	20 (20-25)	20 (20-25)	0.523
**Mineralocorticoid replacement, n (%)**	15 (100)	64 (95.5)	>0.999
Fludrocortisone dose, median (IQR)	150 (100-200)	100 (100-150)	0.654
**DHEA replacement, n (% of women)**	2 (20.0%)	7 (17.1%)	>0.999
**Autoimmune diseases, n (%)**	6 (40.0)	35 (52.2)	0.569
**Charlson Comorbidity Index**			
0, n (%)*	9 (60.0)	19 (28.4)	0.033
1, n (%)	4 (26.7)	11 (16.4)	0.459
≥2, n (%)*	2 (13.3)	37 (55.2)	0.004
**Smoking habit**			
Non-smoker, n (%)	10 (66.7)	55 (82.1)	0.287
Former smoker, n (%)	3 (20.0)	5 (7.5)	0.157
Current smoker, n (%)	2 (13.3)	7 (10.4)	0.666
**Currently working, n (%)***	10 (66.7)	20 (29.9)	0.015
Key workers, n (% of currently working)	3 (30.0)	7 (35.0)	>0.999
Able to work from home, n (% of currently working)	5 (50.0)	12 (60.0)	0.706
**Household arrangement**			
Lives alone, n (%)	2 (13.3)	12 (17.9)	>0.999
Lives with another person, n (%)	4 (26.7)	31 (46.3)	0.245
Lives with more than one person, n (%)	9 (60.0)	24 (35.8)	0.144
**Has the NHS COVID-19 app, n (%)**	7 (46.7)	26 (38.8)	0.576
**Had/planning to have the COVID-19 vaccine, n (%)**	14 (93.3)	65 (97.0)	0.459
**Had/planning to have the flu vaccine this year, n (%)**	14 (93.3)	48 (71.6)	0.101
**Has the flu vaccine every year, n (%)**	13 (86.7)	47 (70.1)	0.333
**Had sick days in the past 12 months, n (%)[b]**	8 (53.3)	37 (55.2)	>0.999
**Had an adrenal crisis in the past 12 months, n (%)[b]**	1 (6.7)	9 (13.4)	0.680
**Had hospital admissions in the past 12 months, n (%)[c]**	2 (13.3)	6 (9.0)	0.634

Abbreviations: COVID-19, Coronavirus disease 2019; IQR, interquartile range; BMI, body mass index; NHS, National Health Service.
[a] Conversion factors: prednisolone 0.25; dexamethasone 0.025.
[b] Excluding sick days / adrenal crisis because of confirmed or suspected COVID-19.
[c] Excluding hospital admissions for adrenal crisis and/or severe COVID-19.
* Significant figures.

Appendix table 4. Clinical characteristics of patients with congenital adrenal hyperplasia with or without COVID-19 diagnosed or suspected.

	**COVID-19 diagnosed or suspected (n=32)**	**No COVID-19 (n=48)**	**p-value**
**Women, n (%)**	22 (68.8)	28 (58.3)	0.480
**Age (years), median (IQR)***	41 (36-52)	36 (26-45)	0.031
**Ethnicity**			
White, n (%)	25 (78.1)	37 (77.1)	>0.999
Asian or Asian British, n (%)	5 (15.6)	9 (18.8)	>0.999
Black, African, Caribbean, or Black British, n (%)	2 (6.3)	2 (4.2)	>0.999
Other ethnic group, n (%)	/	/	
**BMI (kg/m^2), median (IQR)**	25.7 (20.1-31.2)	28.0 (23.5-32.1)	0.382
**Glucocorticoid replacement therapy**			
Hydrocortisone alone – any formulation, n (%)	21 (65.6)	29 (60.4)	0.814
Any other treatment, n (%)	11 (34.4)	19 (39.6)	
Hydrocortisone-equivalent dose, median (IQR)[a]	25 (20-30)	22 (20-25)	0.436
**Mineralocorticoid replacement, n (%)***	17 (53.1)	39 (81.3)	0.012
Fludrocortisone dose, median (IQR)	200 (150-200)	200 (113-200)	0.693
**Charlson Comorbidity Index**			
0, n (%)	21 (65.6)	37 (77.1)	0.311
1, n (%)	5 (15.6)	9 (18.8)	0.773
≥2, n (%)	6 (18.8)	2 (4.2)	0.054
**Smoking habit**			
Non-smoker, n (%)	28 (87.5)	42 (87.5)	>0.999
Former smoker, n (%)	2 (6.3)	4 (8.3)	>0.999
Current smoker, n (%)	2 (6.3)	2 (4.2)	>0.999
**Currently working, n (%)**	15 (46.9)	25 (52.1)	0.820
Key workers, n (% of currently working)	9 (60.0)	12 (48.0)	0.527
Able to work from home, n (% of currently working)	4 (26.7)	13 (52.0)	0.187
**Household arrangement**			
Lives alone, n (%)	5 (15.6)	5 (10.4)	0.510
Lives with another person, n (%)	12 (37.5)	22 (45.8)	0.497
Lives with more than one person, n (%)	15 (46.9)	21 (43.8)	0.822
**Has the NHS COVID-19 app, n (%)**	18 (56.3)	23 (47.9)	0.501
**Had/planning to have the COVID-19 vaccine, n (%)***	30 (93.8)	34 (70.8)	0.020
**Had/planning to have the flu vaccine this year, n (%)**	19 (59.4)	22 (45.8)	0.262
**Has the flu vaccine every year, n (%)**	21 (65.6)	21 (43.8)	0.069
**Had sick days in the past 12 months, n (%)[b]**	19 (59.4)	25 (52.1)	0.647
**Had an adrenal crisis in the past 12 months, n (%)[b]**	2 (6.3)	4 (8.3)	>0.999
**Had hospital admissions in the past 12 months, n (%)[c]**	7 (21.9)	7 (14.6)	0.549

Abbreviations: COVID-19, Coronavirus disease 2019; IQR, interquartile range; BMI, body mass index; NHS, National Health Service.

[a] Conversion factors: prednisolone 0.25; dexamethasone 0.025.

[b] Excluding sick days / adrenal crisis because of confirmed or suspected COVID-19.

[c] Excluding hospital admissions for adrenal crisis and/or severe COVID-19.

* Significant figures.
